# Four year mortality and quality of life after ICU treatment for COVID 19 related acute respiratory distress syndrome

**DOI:** 10.1038/s41598-026-42341-1

**Published:** 2026-03-01

**Authors:** Jacek Zawadzki, Jagoda Kania, Magdalena Murkos, Dominika Zgoła, Anna Noga, Paweł Nowak, Wiktoria Kulińska, Piotr Pawlik, Bartosz Kudliński

**Affiliations:** Department of Anesthesia, Critical Care and Rescue Medicine, Institute of Health Sciences, Collegium Medicum, University of Zielona Góra, 28 Zyty Street, 65-046 Zielona Góra, Poland

**Keywords:** Diseases, Health care, Medical research, Risk factors

## Abstract

Severe COVID-19 leading to ARDS and ICU admission is associated with high early mortality, yet data on long-term outcomes and societal burden remain limited, particularly in Central and Eastern Europe. To describe 4-year mortality, patient-reported functional status and health-related quality of life (HRQoL) among ICU-treated COVID-19 ARDS patients, and to explore early factors associated with short- and long-term mortality as well as long-term recovery. Single-center retrospective–prospective cohort study with structured 4-year telephone follow-up. 283 adults treated in the Temporary ICU Hospital in Zielona Góra, Poland (December 2020–July 2021). Follow-up interviews were completed in 81 of 157 confirmed 4-year survivors. Associations with 30-day mortality and late mortality (among 30-day survivors) were explored using multivariable logistic regression. Survivors completed a structured interview assessing HRQoL (EQ-5D-5 L/EQ-VAS), dyspnoea severity assessed with the mMRC scale, functional status assessed with PCFS, fatigue, brief cognitive screening items, return to work, rehabilitation use, and financial burden. A cumulative post-ICU impairment score (0–6 domains) was constructed. Cost estimates were exploratory and based on public ICU reimbursement rates and patient-reported rehabilitation burden. Thirty-day mortality was 29.0%, and cumulative 4-year mortality was 45%. In adjusted analyses, older age and higher white blood cell count at ICU admission were associated with mortality endpoints (model discrimination up to AUC 0.86, depending on endpoint). Among 4-year survivors, 27.5% reported clinically relevant fatigue, 46.8% insomnia, and a substantial proportion reported persistent limitations across functional and EQ-5D domains. Rehabilitation was reported by 39% and was associated with lower QALY, likely reflecting greater baseline impairment. Median 4-year QALY was 3.7, varying significantly by fatigue, dyspnoea, return-to-work status, and subjective cognitive complaints. Among ICU-treated COVID-19 ARDS patients, long-term mortality remained high and many survivors reported persistent multidomain impairment years after discharge. These findings support structured post-ICU follow-up pathways and targeted rehabilitation and occupational support for long-COVID survivors.

**Supplementary Information:**

The online version contains supplementary material available at 10.1038/s41598-026-42341-1.

## Introduction

The SARS-CoV-2 pandemic may no longer dominate headlines, but its health and societal consequences persist. A large and growing cohort of survivors who experienced critical illness now face long-term sequelae. During peak pandemic waves, up to 15% of infected patients developed respiratory failure requiring advanced respiratory support, including high-flow nasal oxygen (HFNO), non-invasive ventilation (NIV), or invasive mechanical ventilation^1,2^. Despite advances in acute management—including systemic corticosteroids and IL-6 inhibitors—ICU mortality in severe COVID-19 remained substantial, approaching 34% in many reported cohorts^3^. Most follow-up studies have focused on outcomes up to 12 months^4–6^, with fewer extending to 2 or 3 years^7–9^. These studies consistently report persistent dyspnoea, fatigue, and cognitive complaints—features aligned with the post-intensive care syndrome (PICS) framework^10^. However, it remains unclear whether these impairments plateau, worsen, or improve over longer time horizons. Four-year data are scarce, particularly from Central and Eastern Europe, where healthcare access, rehabilitation capacity, and economic safety nets may differ from Western settings^8^. Long-term consequences of ICU-treated COVID-19 should therefore be assessed multidimensionally, including functional status, quality of life, and socioeconomic impact. Many survivors require prolonged sick leave, early retirement, informal caregiving, and incur substantial direct and indirect costs; cost-of-illness estimates can help contextualize the potential value of structured post-ICU follow-up and rehabilitation pathways^11,12^.

### Study aims and outcomes

The primary aim of this study was to describe 4-year survival and health-related quality of life (HRQoL) among patients who required invasive mechanical ventilation for COVID-19-related ARDS in a temporary ICU setting in Poland^13,14^. We pursued four objectives: (i) to explore baseline clinical and laboratory factors associated with early mortality (within 30 days of ICU admission); (ii) to explore factors associated with subsequent mortality among 30-day survivors (late mortality, occurring between day 30 and year 4); (iii) to characterize long-term functional status and patient-reported outcomes among 4-year survivors, including dyspnoea, fatigue, and limitations in daily functioning; and (iv) to estimate HRQoL and quality-adjusted life years (QALYs) using standardized EQ-5D-5 L scoring, alongside an exploratory assessment of indirect societal and financial consequences (rehabilitation burden and return-to-work patterns).

Primary outcomes were all-cause mortality at 30 days and cumulative all-cause mortality at 4 years after ICU admission. In secondary analyses, we assessed late mortality among 30-day survivors. Among 4-year survivors, long-term outcomes included functional status assessed with PCFS, dyspnoea severity assessed with the mMRC scale, and HRQoL derived from EQ-5D-5 L domain scores (including EQ-VAS). Secondary outcomes included subjective cognitive complaints assessed using two screening items adapted from the Cognitive Failures Questionnaire, sleep disturbance (brief screening item), time to return to work, self-reported rehabilitation-related financial burden, and exploratory estimation of indirect costs.

## Materials and methods

### Study design and setting

This study is reported in accordance with the STROBE guidelines for observational cohort studies. We used a retrospective–prospective (ambispective) cohort design. The retrospective component comprised extraction of anonymized demographic, clinical, and laboratory data from electronic medical records for all consecutive adult patients admitted with COVID-19-related ARDS to the Temporary Hospital in Zielona Góra (ICU) between December 2020 and July 2021. The prospective component comprised a structured long-term follow-up conducted in April–August 2025. Survivors were contacted by telephone using the most recent contact details available in hospital records and invited to participate in a physician-administered interview; verbal informed consent was obtained at the start of the call in accordance with the approved protocol. This observational study has been submitted for registration in ClinicalTrials.gov (PRS). The NCT identifier will be added once assigned. The study site was a temporary ICU created exclusively for COVID-19 care in the Lubusz region of Poland.

### Eligibility criteria

All consecutive adult patients admitted to the Temporary Hospital in Zielona Góra between December 2020 and July 2021 with confirmed SARS-CoV-2 infection and COVID-19-related ARDS requiring invasive mechanical ventilation were eligible for inclusion in the baseline cohort. No additional clinical exclusion criteria were applied. Long-term follow-up was offered to patients who were confirmed alive at 4 years after ICU admission; participation in the telephone interview was voluntary. Individuals who declined participation or could not be reached after repeated contact attempts were classified as non-responders for the prospective follow-up component.

### Baseline clinical variables

The baseline variables included demographics (age, sex, body mass index [BMI]), comorbidities (arterial hypertension [AH], coronary heart disease [CHD], chronic kidney disease [CKD], chronic obstructive pulmonary disease [COPD], diabetes mellitus type 2 [DM2], and cancer), vital signs at ICU admission (mean arterial pressure [MAP], heart rate [HR], respiratory rate [RR], Glasgow Coma Scale [GCS]), respiratory parameters (PaO₂/FiO₂ ratio), and laboratory markers (C-reactive protein [CRP], D-dimer, lactate dehydrogenase [LDH], ferritin, creatinine, international normalized ratio [INR], white blood cell count [WBC], and platelet count [PLT]). Admission vital signs were treated as exploratory descriptors, reflecting a single time-point snapshot at ICU presentation.

To better characterize acute illness severity, we additionally included ICU length of stay, APACHE II, and the interval from symptom onset to intubation. Duration of invasive mechanical ventilation was not included in comparative analyses due to substantial missingness. Additional acute-phase treatment and organ support variables were extracted where available, including the presence of shock at admission (vasopressor use and/or MAP < 65; binary), continuous renal replacement therapy (CRRT; binary), and prone positioning (days). Because these variables were incompletely documented, they were used descriptively and interpreted cautiously; analyses were performed on available cases. These severity indicators were reported descriptively and were not incorporated into multivariable modelling.

### Follow-up data collection (telephone interviews)

At four years after ICU discharge, survivors were contacted for a structured telephone interview conducted by a physician using a predefined script. Interviews followed a fixed sequence of questions to ensure consistency. Responses were collected verbally and entered directly into a secure digital form. Interviews typically lasted 10–15 min. No audio recordings were obtained. Participants were contacted on multiple occasions at different times/days; if contact could not be established, they were classified as non-responders for the prospective component.

Vital status at 4 years was ascertained using administrative/electronic records available to the treating center. When contact with a patient was unsuccessful or vital status was unclear, we additionally performed an administrative verification of current healthcare entitlement (eWUŚ) to support further contact attempts; however, eWUŚ was not used as a definitive source of mortality. When vital status remained uncertain, family members were contacted solely to confirm whether the patient was alive or deceased; no additional clinical information was collected from relatives.

### Patient-reported outcomes measures

Patient-reported outcomes covered global functional status, dyspnoea, fatigue, cognitive symptoms, health-related quality of life, and selected social/occupational consequences. Global functional status was assessed with the Post-COVID-19 Functional Status (PCFS) scale (0–4)^15^. Dyspnoea was measured using the modified Medical Research Council (mMRC) scale (0–5)^16^. Health-related quality of life was assessed using the Polish version of the EQ-5D-5 L (five domains rated on a 5-point Likert scale) and the EQ-VAS (0–100). EQ-5D-5 L index values were calculated using the Polish population value set^17,18^. Fatigue was captured using a shortened five-item adaptation based on the Fatigue Assessment Scale, with each item scored from 1 (“never”) to 5 (“always”)^19^. Cognitive symptoms were assessed using two brief screening items adapted from the Cognitive Failures Questionnaire (CFQ), each rated on a 5-point Likert scale^20^; The resulting 2-item CFQ score was used descriptively to capture subjective difficulties with attention and memory; a score ≥ 3 was interpreted as a screen-positive cognitive complaint. Insomnia was assessed using a single yes/no screening question intended to capture patient-perceived sleep disturbance. Additional interview items addressed employment status, time to return to work, disability pension, hospital readmissions, rehabilitation use and duration, and subjective financial burden. Abbreviated or adapted items (fatigue, cognitive symptoms, insomnia) were used descriptively and not as diagnostic tools.

### Economic contextual estimate

For contextual interpretation only (not a formal cost-effectiveness analysis), we performed a simplified cost-per-QALY approximation limited to ICU hospitalization costs. ICU costs were approximated using publicly available Polish estimates of ICU financing per ICU day (assumed range 5000–6000 PLN/day)^21,22^. The median ICU length of stay among long-term survivors was used to derive an approximate per-patient ICU cost, which was contrasted with the median 4-year QALY observed among follow-up responders. This contextual estimate does not include post-ICU healthcare utilization, rehabilitation expenditure, or indirect societal costs.

### Statistical analysis

Continuous variables are presented as medians with interquartile ranges (IQR) and were compared using the Wilcoxon rank-sum test. Categorical variables are presented as counts and percentages and were compared using the chi-squared test or Fisher’s exact test, as appropriate. For 30-day mortality, we performed univariable logistic regression to screen candidate variables and fitted parsimonious multivariable logistic regression models, reporting odds ratios (ORs) with 95% confidence intervals (CIs). Model discrimination was evaluated using the area under the receiver operating characteristic curve (AUC), and calibration was assessed with the Hosmer–Lemeshow goodness-of-fit test. Adjusted associations were visualized using forest plots (Supplementary Figs. S1–S3). An analogous logistic approach was applied to late mortality (death between 30 days and 4 years among 30-day survivors), treating the outcome as a predefined binary endpoint.

Because APACHE II is a composite severity score, it was not modelled together with its component physiological and laboratory variables to avoid collinearity and overfitting—when used, APACHE II served as the primary indicator of acute illness severity. For 4-year survivors, descriptive statistics were used to summarize long-term functional status and social outcomes. Comparisons of quality-adjusted life years (QALYs) were performed using the Wilcoxon rank-sum test. QALYs were estimated by multiplying the EQ-5D-5 L index value by a four-year time horizon. Functional status assessed with PCFS was not included in multivariable modelling due to the very small number of patients with moderate or greater limitation (*n* = 3). Potential multicollinearity was assessed using variance inflation factors (VIFs). All analyses were performed using Stata version 19 (StataCorp LLC, College Station, TX, USA). A two-sided *p* < 0.05 was considered statistically significant.

### Ethics

The study was approved by the Bioethics Committee of the University in Zielona Góra, Poland (ref. 03/165/2021). The study comprised two components with different consent requirements. First, the retrospective ICU-phase component used routinely collected electronic medical record data; the Committee granted a waiver of written informed consent due to minimal risk and the use of pseudonymized (coded) data. No additional procedures or patient contact occurred for this record-review component; therefore, no patient or proxy consent was sought or required. Second, the prospective component consisted of structured 4-year telephone follow-up interviews; the Committee approved the use of verbal informed consent. Prior to the interview, participants received standardized study information and provided verbal consent, which was documented in the study database; no audio recordings were obtained. Individuals who were deceased, could not be contacted, or declined participation contributed only to the retrospective ICU-phase dataset under the waiver described above. Data were processed in compliance with the General Data Protection Regulation (GDPR). All methods were carried out in accordance with relevant guidelines and regulations, including the Declaration of Helsinki.

## Results

### Acute outcomes (30-day mortality)

**Fig. 1:**
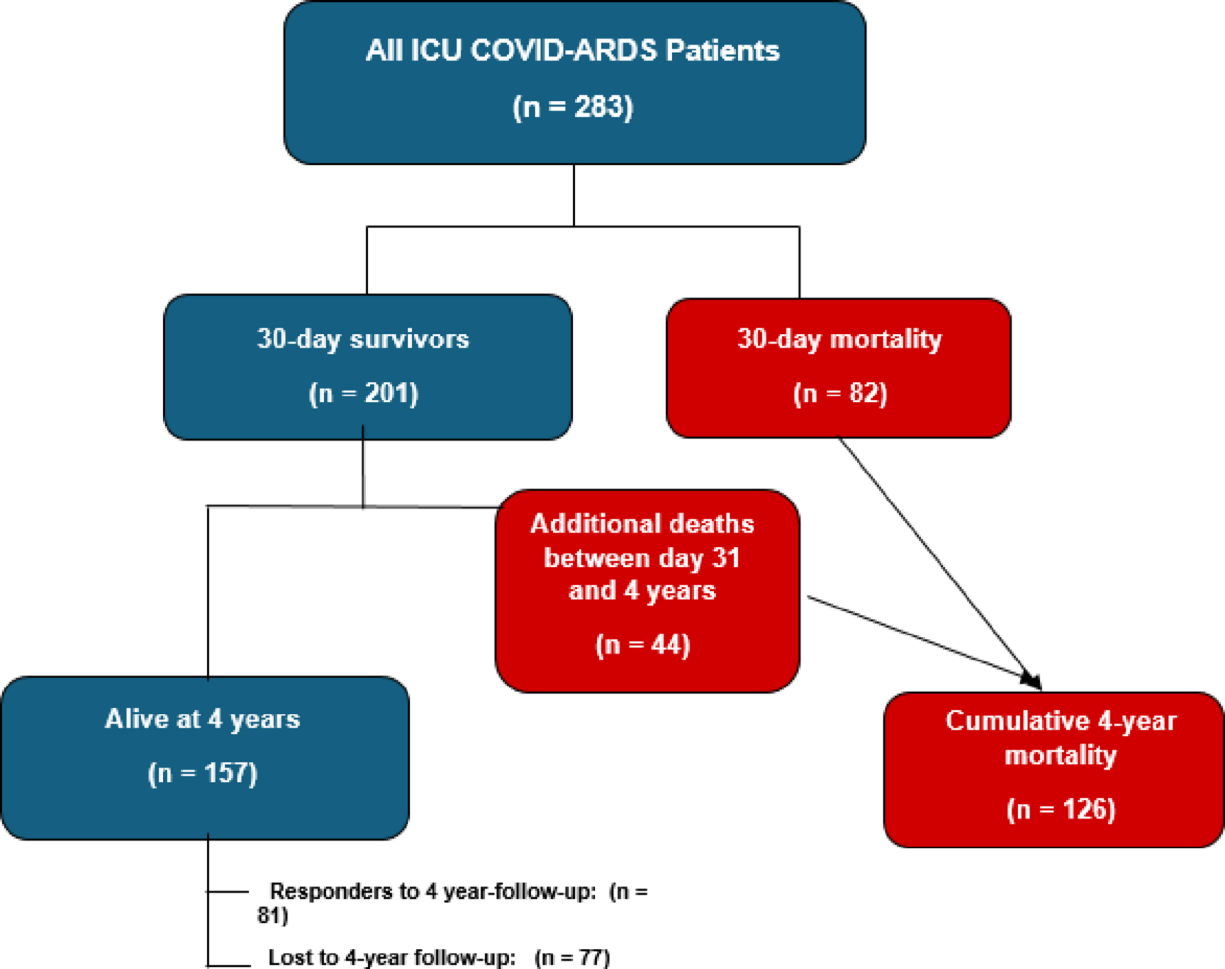
Flow diagram-inclusion, follow-up, and survival over 4 years. Study flowchart illustrating cohort derivation, early and late all-cause mortality, and long-term follow-up. Absolute numbers (n) are shown at each stage. Cumulative 4-year mortality includes deaths occurring both within the first 30 days and between 30 days and 4 years after ICU admission. Patients who died were not eligible for follow-up; only 4-year survivors were contacted for telephone interviews.

A total of 283 patients with COVID-19–related ARDS were treated in the ICU during the study period. Of these, 201 (71.0%) survived at least 30 days after ICU admission, whereas 82 (29.0%) died within the first 30 days. Among the 30-day survivors, 44 additional deaths occurred during follow-up, corresponding to 21.9% (44/201) of early survivors (and 15.5% of the full cohort). Overall, cumulative 4-year all-cause mortality was 44.5% (126/283). The patient flow and denominators for early and late deaths, as well as long-term follow-up participation, are shown in Fig. 1. Baseline clinical and laboratory characteristics by 30-day survival status are summarized in Table 1.

**Table 1:** Baseline characteristics by 30-days survival status.

Variable Continous	30-days survivors *n* = 201 Mean ± SD Median [IQR]*	Non-survivors *n* = 82 Mean ± SD Median [IQR]*	*p*-value
Age (years)	57.8 ± 10.7	62.5 ± 9	< 0.05
WBC (10^3^/µL)	10.8 ± 5.4	12.6 ± 6.7	< 0.05
LDH (u/L)	606 ± 230.8	710.7 ± 305.7	< 0.05
PLT (10^3^/µL)	310.7 ± 130.3	268 ± 124.7	< 0.05
Creatinine (mg/dL)	1.08 ± 0.97	1.1 ± 0.69	0.73
BUN (mg/dL)	28.6 ± 43	30.3 ± 34.5	0.76
PFR (PaO₂/FiO₂)	117.7 ± 81.1	110.6 ± 68.2	0.49
CRP (mg/L)	140.1 ± 98.2	159.4 ± 97.1	0.13
D-dimer (µg/L)*	1489.5 [885–4385]	2507 [959–13951]	< 0.05
Ferritin (ng/mL)*	1563.1 [750–2000]	1914.8 [1054–2000]	0.053
APACHE II score (points)	9 [7–12]	12 [9–14]	< 0.005
ICU length of stay (days)	9 [6–15]	11 [8–17]	< 0.05
Time from symptom onset to intubation (days)	2 [1–4]	2 [1–5]	0.95
Prone positioning (days)	5 [3–7]	5 [3–8]	0.7
Binary	n (%)	n (%)	
Prone positioning	148 (93)	59 (91)	0.55
Female sex	70 (34.8)	26 (31.7)	0.62
Shock at admission to ICU	40 (22.1)	20 (29.4)	0.23
CRRT required	9 (8.2)	10 (20.4)	< 0.05
Coronary heart disease	19 (9.5)	8 (9.9)	0.92
Arterial hypertension	105 (52.2)	49 (59.8)	0.25
Chronic kidney disease	7 (3.5)	1 (1.2)	0.3
COPD	7 (3.5)	3 (3.7)	0.93
Active cancer	12 (6)	3 (3.7)	0.44
Current smoker	13 (6.5)	6 (7.4)	0.78
Type 2 diabetes	34 (17)	22 (27.5)	< 0.05

Comparison of demographic, clinical, and biochemical parameters between 30-day survivors and non-survivors. Continuous variables are reported as mean ± SD or median [IQR], depending on distribution; categorical variables as n (%). Continuous variables were compared using Student’s t-test or the Mann–Whitney U test, as appropriate; categorical variables were compared using the chi-squared test or Fisher’s exact test. Values above 2000 ng/mL were reported as “>2000” by the laboratory; thus, upper quartiles may be right-censored. Analyses were performed on available cases; missingness was present for shock (vasopressor use and/or MAP < 65 at admission) (*n* = 34), CRRT (*n* = 124), and prone positioning duration (days prone, *n* = 59). Abbreviations: BUN, blood urea nitrogen; WBC, white blood cell count; PLT, platelet count; COPD, chronic obstructive pulmonary disease; LDH, lactate dehydrogenase; PFR, PaO_2_/FiO_2_ ratio; CRRT, continuous renal replacement therapy. Oxygenation points in APACHE II were calculated using PaO_2_ rather than A–aDO_2_ in some cases due to incomplete FiO_2_/PaCO_2_ data, which may slightly underestimate physiologic severity.

Patients who died within 30 days were older (62.5 ± 9.0 vs. 57.8 ± 10.7 years; *p* < 0.05) and had higher baseline LDH (710.7 ± 305.7 vs. 606.0 ± 230.8 U/L; *p* < 0.05), D-dimer (2507 [959–13951] vs. 1489.5 [885–4385] µg/L; *p* < 0.05), and WBC (12.6 ± 6.7 vs. 10.8 ± 5.4 × 10^3^/µL; *p* < 0.05), with lower platelet counts (268 ± 124.7 vs. 310.7 ± 130.3 × 10^3^/µL; *p* < 0.05) compared with 30-day survivors (Table 1). Type 2 diabetes was also more prevalent among non-survivors. No significant differences were observed for creatinine, CRP, ferritin, BUN, PaO_2_/FiO_2_ ratio, or most other comorbidities. Indicators of acute illness severity also differed between groups: non-survivors had higher APACHE II scores (12 [9–14] vs. 9 [7–12]; *p* < 0.005) and longer ICU length of stay (11 [8–17] vs. 9 [6–15] days; *p* < 0.05). Time from symptom onset to intubation did not differ (2 [1–5] vs. 2 [1–4] days; *p* = 0.945).

**Fig. 2:**
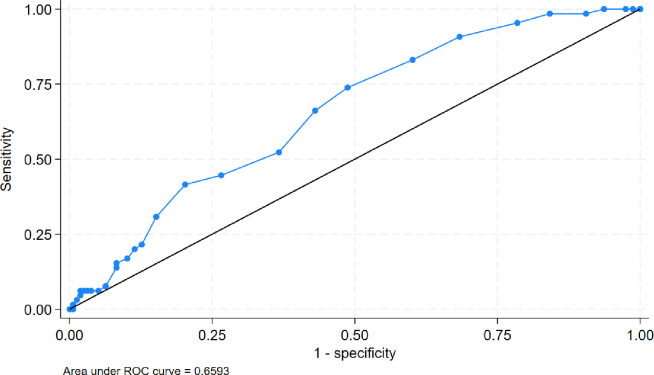
Receiver operating characteristic (ROC) curve for the final multivariable model predicting 30-day mortality based on APACHE II score. The model demonstrated moderate discrimination (AUC 0.66, 95% CI 1.03–1.14).

An exploratory multivariable biomarker-based model including age, LDH, D-dimer, WBC, and platelet count showed good apparent discrimination for 30-day mortality (AUC 0.76, 95% CI 0.69–0.83; Supplementary Fig. S4). However, given correlation among laboratory predictors and the risk of overfitting, a more parsimonious model based on APACHE II was selected as the primary discrimination model (AUC 0.66; Fig. 2).

In sensitivity (complete-case) analyses additionally including shock proxy (vasopressor use and/or MAP < 65), CRRT use, and prone positioning (any) -—ge and WBC remained the only variables independently associated with 30-day mortality, and the organ-support variables were not statistically significant. These estimates should be interpreted cautiously because documentation of these interventions was incomplete, resulting in reduced sample size.

**Table 2:** Factors independently associated with 30-day mortality in patients with COVID-19-related ARDS.

Variable	OR	95% CI	*p*-value
APACHE II score (points)	1.04	0.98–1.1	0.19
Age (years)	1.05	1.01–1.08	**< 0.05**
WBC (10^3^/µL)	1.07	1.01–1.14	**< 0.05**
ICU length of stay (days)	1.01	0.97–1.04	0.48

Multivariable logistic regression examining factors associated with 30-day mortality. Results are presented as adjusted odds ratios (aORs) with 95% confidence intervals (CIs). Variables were selected based on clinical relevance and univariate associations, with preference given to composite severity measures to reduce collinearity and overfitting. WBC, white blood cell count.

In multivariable logistic regression, older age (adjusted OR 1.05 per year, 95% CI 1.01–1.08; *p* = 0.01) and higher WBC (adjusted OR 1.07, 95% CI 1.01–1.14; *p* = 0.02) were independently associated with 30-day mortality, whereas APACHE II was not statistically significant after adjustment (*p* = 0.19), indicating substantial overlap in prognostic information with age and inflammatory burden (Table 2).

### Long-term outcomes (4-year mortality)

Among the 201 patients who survived the first 30 days after ICU admission, 44 died during the subsequent follow-up period (day 30 to year 4). As shown in Table 3, late non-survivors were older (63.5 ± 9.6 vs. 56.2 ± 10.5 years, *p* < 0.05) and, in unadjusted comparisons, had a generally more severe acute-phase profile, including higher admission LDH, D-dimer, troponin, creatinine, WBC, PCT and PaCO_2_, along with lower GCS and platelet counts. Coronary heart disease, chronic kidney disease and COPD were also more frequent among late non-survivors. ICU length of stay did not differ significantly between groups.

**Table 3:** Comparison of baseline characteristics between long-term survivors and patients with late mortality (30 days to 4 years after ICU admission).

Variable Continous	Long-term survivors *n* = 157 Mean ± SD Median [IQR]*	Long-term non-survivors *n* = 44 Mean ± SD Median [IQR]*	*p*-value
Age (years)	56.2 ± 10.5	63.5 ± 9.6	< 0.05
WBC (10^3^/µL)	10.3 ± 5	12.7 ± 6.5	< 0.05
Creatinine (mg/dL)	0.96 ± 0.65	1.6 ± 1.7	< 0.05
LDH (u/L)	587.1 ± 227.6	703.7 ± 226.5	< 0.05
PLT (10^3^/µL)	327 ± 132.5	250 ± 101.7	< 0.05
PaCO2 (mmHg)	42.8 ± 12.8	48.1 ± 18.6	< 0.05
GCS (pts)	13.7 ± 3.6	11.4 ± 5.3	< 0.05
Heart Rate (bpm)	87.8 ± 18.2	93.8 ± 21.7	0.07
Respiratory Rate (rpm)	24.8 ± 11.8	29.5 ± 13.4	0.08
CRP (mg/L)	134 ± 99.1	161.7 ± 92.7	0.1
BUN (mg/dL)	27.5 ± 46.6	33.3 ± 22.1	0.46
PaO2 (mmHg)	87.8 ± 41.4	81.5 ± 45.4	0.39
D-dimer (µg/L)*	1381 [836–2802]	3567 [1413–12 103]	< 0.05
Troponin (ng/mL)*	10 [4.4–27.2]	55.6 [17.6–521.4]	< 0.05
PCT (ug/L)*	0.18 [0.09–0.38]	0.33 [0.18–1.3]	< 0.05
Ferritin (ng/mL)*	1553.7 [712–2000]	1894.8 [1171–2000]	0.24
PFR (PaO_2_/FiO_2_)*	92.5 [71–154]	73 [61–110]	0.08
APACHE II score (points)	9 [6–12]	13,5 [12–20]	< 0.001
ICU length of stay (days)	9 [6–14]	8,5 [3–18]	0.49
Binary	n (%)	n (%)	
Coronary heart disease	8 (5.1)	11(25)	< 0.05
Chronic kidney disease	1 (0.6)	6 (13,6)	< 0.05
COPD	3 (1.9)	4 (9.3)	< 0.05
Current smoker	10 (6.4)	3 (6.8)	0.92
Arterial hypertension	81(51.6)	24 (54.5)	0.73
Type 2 diabetes	26 (16.6)	8 (18.6)	0.75
Active cancer	10 (6.3)	2 (4.8)	0.7
Female sex	102 (65)	29 (66)	0.91

Baseline characteristics of 30-day survivors stratified by late mortality (death between day 31 and year 4). Continuous variables are reported as mean ± SD for approximately normally distributed variables and as median [IQR] for skewed variables, and were compared using Student’s t-test or the Wilcoxon rank-sum test, as appropriate. Categorical variables are presented as n (%) and were compared using the chi-squared test or Fisher’s exact test. *Skewed variables are presented as median [IQR]. For laboratory parameters with upper reporting limits, upper quartiles may be right-censored. GCS, Glasgow Coma Scale; PaCO_2_, arterial partial pressure of carbon dioxide; PaO_2_, arterial partial pressure of oxygen; BUN, blood urea nitrogen; LDH, lactate dehydrogenase; WBC, white blood cell count; PLT, platelet count; COPD, chronic obstructive pulmonary disease; CRP, C-reactive protein; PCT, procalcitonin; PFR, PaO_2_/FiO_2_ ratio.

Table 3. 

**Table 4:** Factors independently associated with late mortality (30 days to 4 years) after ICU admission.

Variable	OR	95% CI	*p*-value
Age (years)	1.08	1.07–1.13	**< 0.05**
ICU length of stay (days)	1.02	0.98–1.06	0.34
Type 2 diabetes	1.06	0.41–2.75	0.9

Multivariable logistic regression for late mortality (death between day 31 and year 4) among 30-day survivors. Adjusted odds ratios (ORs) with 95% confidence intervals (CIs) are presented. The model was specified a priori to emphasize baseline patient characteristics and longer-term trajectory rather than acute severity composites. The multivariable model was estimated using complete-case data (*N* = 198; late deaths = 41), due to missingness in covariates. Two-sided p-value < 0.05 was considered statistically significant.

In multivariable logistic regression, older age remained the only factor associated with late mortality (aOR 1.06 per year, 95% CI 1.02–1.10; *p* < 0.05), while ICU length of stay and type 2 diabetes were not significant after adjustment (Table 4).

Baseline characteristics of responders and patients lost to follow-up are shown in Supplementary Table S1. Groups were broadly similar for available baseline variables. However, residual selection bias cannot be excluded, particularly due to incomplete availability of some severity indicators and unmeasured social factors.

### Functional, quality-of-life, and socio-economic outcomes in 4-year survivors

Among follow-up responders (Table 5), 19/81 (30%) reported any functional limitation on the PCFS scale (≥ 1), including 3/81 (3.7%) with moderate or worse limitation (≥ 3). Insomnia was reported by 37/79 (46.8%). Clinically relevant fatigue (FAS ≥ 12/25) was present in 22/80 (27.5%). Approximately one in five participants reported moderate or severe pain/discomfort on EQ-5D-5 L, and 7/46 (15.2%) of those employed pre-COVID had not returned to full-time work. Rehabilitation was reported by 31/79 (39.2%), and 30/80 (37.5%) reported at least one rehospitalization during follow-up. The estimated median QALY at 4 years was 3.7 [3.3–3.9].

**Table 5:** Functional, psychological, and socioeconomic outcomes among follow-up responders (*n* = 81).

1. Functional status and quality of life	
Variable *n* = 81 (follow up responders)	Value
PCFS score (0–4): any limitation (≥ 1), n (%)	19 (30)
PCFS score (0–4): moderate or worse (≥ 3), n (%)	2 (3.4)
EQ-5D: Mobility (1–5): any limitation (≥ 2), n (%)	13 (16.3)
EQ-5D: Mobility (1–5): moderate or worse (≥ 4), n (%)	6 (7.5)
EQ-5D: Self-care(1–5): any limitation (≥ 2), n (%)	8 (11.3)
EQ-5D: Self-care(1–5): moderate or worse (≥ 4), n (%)	2 (2.8)
EQ-5D: Usual activities(1–5): any limitation (≥ 2), n (%)	11 (13.8)
EQ-5D: Usual activities(1–5): moderate or worse (≥ 4), n (%)	6 (7.5)
EQ-5D: Pain/discomfort(1–5): any limitation (≥ 2), n (%)	38 (47.5)
EQ-5D: Pain/discomfort(1–5): moderate or worse (≥ 4), n (%)	15 (21.3)
EQ-5D: Anxiety/depression(1–5): any limitation (≥ 2), n (%)	23 (29.1)
EQ-5D: Anxiety/depression(1–5): moderate or worse (≥ 4), n (%)	16 (20.3)
EQ-5D : visual analogue scale (VAS) (0-100), median [IQR]	70 [60–80]
2. Fatigue Assessment Scale—Modified (FAS)	
Variable	Value
FAS total score (5–25), median [IQR]	8.5 [5–12]
FAS ≥ 12/25 (clinically relevant fatigue), n (%)	22 (27.5)
3. Cognition and sleep	
Variable	Value
Subjective cognitive complaint 1 (CFQ1 ≥ 3), n (%)	31 (39.2)
Subjective cognitive complaint 1 (CFQ2 ≥ 3), n (%)	31 (39.2)
Insomnia, n (%)	37 (46.8)
4. Employment and rehabilitation	
Variable n (%)	Value
Employed pre-COVID: Full time job or self-employed, n (%)	46 (58.2)
Retired or pensioned, n (%)	32 (40.5)
Unemployed, n (%)	1 (1.3)
Did not return to full-time work among pre-COVID employed, n (%)	7 (15.2% of pre-COVID)
Returned to full-time work among pre-COVID employed, n (%)	39 (84.8% of pre-COVID)
Time to return to full-time work among pre-COVID employed (months), median [IQR]	3.5 [2–6]
Any rehabilitation received, n (%)	31 (39.2)
No rehabilitation, n (%)	48 (60.8)
Duration of rehabilitation among rehabilitated patients (days), median [IQR]	35 [28–56]
Rehab debts, n (%)	4 (5)
Hospitalized again post-COVID, n (%)	30 (37.5)
5. QALY	
Estimated QALY at 4 years, median [IQR]	3.7 [3.3–3.9]

PCFS, Post-COVID Functional Status scale score ≥ 3 interpreted as relevant; EQ-5D, EuroQol-5 Dimension instrument; FAS, Modified 5-item Fatigue Assessment Scale (range 5–25); ≥12 considered clinically significant fatigue. CFQ: 2-item version of the Cognitive Failures Questionnaire; score ≥ 3 interpreted as relevant cognitive complaint. Insomnia was defined by self-reported answer to the question “Do you currently suffer from insomnia?” (yes/no). QALY: quality-adjusted life-years over a 4-year period. Values are presented as mean (SD), median [IQR], or number (%), as appropriate.

Patients with clinically relevant fatigue (FAS ≥ 12/25), cognitive complaints (CFQ ≥ 3), clinically relevant dyspnoea assessed with the mMRC scale (mMRC ≥ 3), and those who did not return to full-time work had lower QALY at 4 years (Table 6). QALY did not differ by insomnia status. Participants who reported rehabilitation also had lower QALY, likely reflecting confounding by indication (i.e., rehabilitation use as a marker of greater symptom burden).

**Table 6:** Factors associated with QALY at 4-year follow-up.

Variable	Reference Group A *n* (patients)	Exposed Group B *n* (patients)	Median QALY A	Median QALY B	*p* value
Fatigue (FAS ≥ 12/25)	FAS < 12 (*n* = 58)	FAS ≥ 12 (*n* = 22)	3.9	3.3	< 0.05
Insomnia	No (*n* = 42)	Yes (*n* = 37)	3.7	3.7	0.12
Cognitive complaint (CFQ ≥ 3)	No/mild cognitive issue (*n* = 48)	Relevant cognitive issue (*n* = 31)	3.9	3.3	< 0.05
Return to work after ICU	Yes (*n* = 39)	No (*n* = 7)	3.9	2.9	< 0.05
Rehabilitation	No (*n* = 49)	Yes (*n* = 31)	3.7	3.5	< 0.05
Dyspnoea (mMRC ≥ 3)	No (*n* = 68)	Yes (*n* = 11)	3.7	3.3	< 0.05

Factors associated with QALY at 4-year follow-up. Median QALY values within each subgroup are presented. Group A served as the reference (better outcome or absence of exposure), while Group B represents the exposed or impaired category. Comparisons were performed using the Mann–Whitney U test. QALY was estimated as the EQ-5D-5 L index value multiplied by a 4-year survival time horizon. Subgroup sizes (n) are shown for each comparison and may vary due to item-level missingness and because some variables apply only to specific subsets (e.g., return to work among pre-COVID employed participants).

Collinearity between the six exposure variables was low (mean VIF 1.29). Therefore, these variables were entered simultaneously in an exploratory multivariable model presented in the Supplementary Materials (Fig. S3). The resulting estimates should be interpreted as associations rather than causal effects.

We additionally constructed a simple cumulative impairment score (0–6). In exploratory analysis, the score showed good discrimination for lower versus higher 4-year QALY (≤ 3.5) (AUC 0.81, 95% CI 0.71–0.91). A threshold of ≥ 3 yielded sensitivity 58%, specificity 89%, and LR + 5.5, correctly classifying 77% of participants.

## Discussion

### Principal findings

In this retrospective–prospective cohort of 283 patients with COVID-19-related ARDS treated with invasive mechanical ventilation, mortality remained high both early and long after ICU admission. Thirty-day all-cause mortality was 29% (82/283). Among 30-day survivors (*n* = 201), a further 44 deaths occurred between day 30 and year 4, resulting in a cumulative 4-year mortality of 45% (126/283). In the acute phase, death within 30 days was associated with older age and a greater inflammatory burden at ICU admission. Several admission laboratory and clinical parameters differed between survivors and non-survivors, but many of these measures were correlated, indicating that they capture overlapping aspects of acute illness severity rather than distinct independent signals. Among patients who survived beyond 30 days, late mortality up to 4 years was most consistently associated with older age, suggesting that long-term survival after severe COVID-19 depends not only on the acute episode but also on underlying patient-related vulnerability. Of the 157 patients alive at 4 years, 81 completed the telephone follow-up. Respondents reported a substantial burden of persistent symptoms and perceived functional limitations. Fatigue, sleep disturbance, cognitive complaints, dyspnoea, and limitations in daily activities were common, and a meaningful proportion of survivors reported moderate or worse impairment in at least one functional domain. In addition, over 15% of previously employed participants had not returned to full-time work, and more than one third reported at least one re-hospitalisation during follow-up. Finally, we explored a simple cumulative impairment score based on six post-ICU sequelae. A higher number of concurrent impairments was associated with lower long-term quality-adjusted life years, underscoring that recovery after ICU-treated COVID-19 is multidimensional and that symptom clustering may be more informative than any single complaint.

### Early mortality and acute-phase predictors

In multivariable logistic regression, older age and higher white blood cell count (WBC) at ICU admission were independently associated with 30-day all-cause mortality, consistent with reports identifying age and inflammatory activation as key early risk correlates in severe COVID-19^23–25^. Other admission biomarkers (LDH, D-dimer, platelet count) showed univariable associations with mortality, but their effects attenuated after adjustment, likely reflecting shared pathophysiology and intercorrelation among acute-phase abnormalities in COVID-19-related ARDS^26,27^. We also examined markers of acute severity and organ-support exposure. Although APACHE II was strongly associated with 30-day mortality in univariable analysis, it was not statistically significant after adjustment for age and WBC, suggesting that in our cohort much of the prognostic information captured by this composite score overlapped with baseline vulnerability and inflammatory burden. This is clinically plausible and aligns with prior ICU COVID-19 cohorts where age and acute severity are dominant drivers of early mortality risk^28–30^. In sensitivity (complete-case) analyses incorporating a shock proxy (vasopressor use and/or MAP < 65 mmHg), prone positioning, and renal replacement therapy, associations for age and WBC were directionally consistent, whereas organ-support variables did not retain independent statistical significance. These findings should be interpreted cautiously due to substantial missingness and reduced power for adjusted modelling. Finally, traditional respiratory severity descriptors (PaO_2_/FiO_2_ and Berlin ARDS category) were not independently associated with early mortality, potentially reflecting restricted variability at ICU admission in a cohort dominated by at least moderate ARDS requiring invasive ventilation. Chronic comorbidities showed only modest univariable differences and did not retain independent associations after adjustment, consistent with registry data suggesting that demographics and acute illness severity may outweigh comorbidity burden in explaining short-term ICU mortality in COVID-19^31^.

### Late mortality, and long-term predictors

Among 30-day ICU survivors, late non-survivors differed from long-term survivors at baseline: they were older and more frequently presented with markers of acute physiological derangement and organ dysfunction at ICU admission, and they experienced a more severe acute course (e.g., higher APACHE II and differences in organ-support exposure where documented). Together, these patterns support the concept that long-term survival after ICU-treated COVID-19 ARDS reflects both patient-level vulnerability and the intensity of the index critical illness^32,33^. In multivariable analysis of late mortality (deaths between day 30 and year 4 among 30-day survivors), age was the only variable that remained independently associated with late mortality (Table 4). This suggests that, conditional on surviving the acute phase, residual long-term risk is predominantly driven by baseline vulnerability, whereas individual admission biomarkers or isolated ICU interventions provide limited incremental prognostic information in adjusted models. A similar predominance of simple baseline features has been observed in large COVID-19 prognostic cohorts, where age consistently retained robust associations after adjustment^23,34^. These adjusted late-mortality analyses should be interpreted cautiously: event counts were limited and several organ-support variables were incompletely documented during surge conditions, reducing power and potentially attenuating independent associations. We therefore consider these findings exploratory and hypothesis-generating.

### Health-related quality of life and QALY in 4-year survivors

Beyond survival, we assessed long-term patient-reported functional status and HRQoL using structured follow-up. Among responders with available EQ-5D-5 L data (*n* = 83), the median 4-year QALY was 3.7 (IQR 3.3–3.9), suggesting that many reachable survivors reported relatively preserved long-term HRQoL. These findings should be interpreted cautiously due to survivorship and response bias, and because PROMs capture perceived rather than objectively measured functioning; nevertheless, they provide clinically meaningful insight into post-ICU recovery^35–37^. QALY values differed across patient-centered recovery domains, including return-to-work status and symptom burden (e.g., fatigue and dyspnoea), underscoring the multidimensional nature of survivorship after ICU-treated ARDS^35–38^. For contextual interpretation only, we additionally report an exploratory ICU-only cost-per-QALY approximation based on publicly available Polish estimates of ICU per-diem financing and the median ICU length of stay in our cohort. This is not an incremental cost-effectiveness analysis and does not include downstream healthcare utilization, rehabilitation, or indirect societal costs^39^. When contrasted with the official Polish cost-effectiveness threshold (currently 244,821 PLN/QALY), the resulting ICU-only ratio remained substantially below this benchmark^40^.

### Rehabilitation

Only 39% of surveyed survivors reported participation in any rehabilitation during the four years after ICU discharge, despite rehabilitation being commonly recommended following critical illness. Limited uptake likely reflects structural constraints, including workforce shortages and service accessibility barriers. In line with this, recent WHO reports indicate that Poland remains below the EU average in rehabilitation workforce capacity and selected system-level indicators relevant to service availability^41,42^. Interestingly, patients who reported rehabilitation had slightly lower QALY than those who did not (3.5 vs. 3.7; *p* < 0.05). This pattern is most plausibly explained by confounding by indication, whereby patients with greater functional impairment are more likely to be referred to, or seek, rehabilitation rather than reflecting harm from rehabilitation itself. One respondent also described a perceived mismatch between program intensity and individual needs, highlighting the importance of patient-tailored pathways. Overall, evidence supports rehabilitation as an important component of recovery after mechanical ventilation and severe COVID-19, but our findings suggest that access and alignment with patient needs remain key implementation challenges^43,44^.

### Persistent symptoms and functional limitations

Despite generally favorable QALY among 4-year survivors, a substantial subgroup reported persistent physical and psychological symptoms. Clinically significant fatigue (FAS ≥ 12) affected 27.5% of respondents and was independently associated with lower QALY, consistent with prior reports indicating long-term fatigue in a sizeable proportion of ICU survivors, including those recovering from COVID-19–related ARDS^45,46^. Fatigue is a core feature of post-intensive care syndrome (PICS) and likely reflects a multifactorial burden spanning neuromuscular impairment, ongoing inflammation, and psychological stress^46^. Insomnia was reported by 46.8% of respondents, placing our estimate toward the upper range described in post-COVID and long-COVID cohorts^47,48^. This high prevalence may reflect a combination of post-critical illness stress, neurobiological disruption, and limited access to structured post-ICU follow-up. Although 29% of patients reported symptoms of anxiety or depression, many did not explicitly attribute insomnia or mood symptoms to ICU-related psychological trauma, despite frequently describing the ICU experience as highly distressing. This apparent dissociation aligns with evidence linking post-ICU psychological morbidity to chronic sleep impairment in critical illness survivors^49^.

### Predicting reduced long-term QALY: a simple post-ICU score

In univariable analyses, several post-ICU domains (fatigue, insomnia, subjective cognitive complaints, persistent dyspnoea, rehabilitation use, and not returning to full-time work) were associated with lower 4-year QALY. However, in a multivariable model including all domains simultaneously, no single domain remained statistically significant, likely reflecting limited power in a modest follow-up sample and substantial clustering of post-ICU impairments, which inflates uncertainty in adjusted estimates. We therefore interpret these findings as exploratory. To better capture overall survivorship burden, we summarized impairments using a simple cumulative count (0–6), consistent with multidimensional post-ICU frameworks (including PICS) and prior work highlighting long-term cognitive and socioeconomic sequelae after critical illness and ARDS^46,50,51^. The proposed score is intended as a descriptive research tool and requires external validation before any consideration of clinical use.

### Strenghts, limitations and personal involvement

This study provides one of the longest follow-up assessments of ICU-treated COVID-19–related ARDS reported from Central and Eastern Europe. By linking routinely collected acute-phase ICU data with structured 4-year follow-up interviews, we were able to describe mortality over time and long-term patient-reported health status in a well-defined cohort treated during a pandemic surge. The inclusion of patient-reported functional and HRQoL outcomes adds clinically meaningful information beyond survival and reflects the multidimensional burden of ICU survivorship.

Several limitations should be considered. The study was ambispective: baseline ICU variables were extracted retrospectively from routine electronic records, whereas long-term outcomes were collected prospectively by telephone. Long-term outcomes relied on PROMs and therefore reflect perceived rather than objectively measured functioning; recall bias is likely limited for current status measures (e.g., EQ-5D-5 L, mMRC, PCFS) but may affect retrospectively reported items such as rehabilitation exposure and financial burden. Documentation during surge conditions was incomplete for several acute-phase variables, limiting their inclusion in adjusted analyses (e.g., APACHE II available for 78.8%, symptom-onset–to-intubation interval for 58.7%, and mechanical ventilation duration missing for most participants). Similarly, organ-support variables (e.g., vasopressors as a shock proxy, CRRT, and prone positioning duration) were incompletely captured, reducing power for multivariable modelling and potentially introducing bias if missingness was not random. The modest response rate to 4-year follow-up raises the possibility of selection bias related to social factors, migration, or inability to establish contact. The absence of a control group (e.g., non-ICU COVID-19 or non-COVID ARDS survivors) limits generalizability, and formal neurocognitive or psychiatric testing was not available. Given the number of candidate predictors relative to event counts and the lack of external validation, multivariable analyses and the proposed post-ICU impairment score should be interpreted as exploratory.

Investigator involvement warrants transparent reporting. Follow-up interviews were conducted using a predefined script with direct entry into a secure digital form. When a participant could not be reached and vital status remained uncertain, hospital administrative information was used to guide additional contact attempts; in selected cases, administrative verification of healthcare entitlement (eWUŚ) was used as an auxiliary step, recognizing that it is not a mortality registry. If uncertainty persisted, family members were contacted only to confirm whether the patient was alive or deceased, without collecting additional clinical information. Misclassification of vital status cannot be fully excluded, particularly for non-residents or individuals who left Poland. All follow-up interviews were conducted by the principal investigator (JZ), who had been involved in the clinical care of a proportion of the cohort during the index ICU admission. This continuity may have facilitated engagement, but it may also introduce interviewer and social desirability bias.

### Implications for clinical practice and policy

Our findings suggest several practical considerations for post-ICU care after severe COVID-19. First, routinely available admission variables (such as older age and inflammatory markers) were associated with early and late mortality in our cohort and may be useful for contextual clinical communication and prioritizing follow-up planning, rather than as stand-alone prognostic tools. Second, the high prevalence of patient-reported long-term symptoms—including fatigue, sleep disturbance, dyspnoea, cognitive complaints, and limitations in daily activities—supports the need for structured, multidisciplinary follow-up pathways that include symptom screening, functional assessment, and return-to-work counselling. Third, the relatively low uptake of rehabilitation and patient-reported financial burden indicate potential access barriers; therefore, policy efforts should focus on improving availability and geographic accessibility of rehabilitation services and reducing out-of-pocket costs. Overall, post-ICU pathways should address physical, cognitive, and socioeconomic domains, reflecting the multidimensional consequences of critical illness.

## Conclusions

In this single-center retrospective–prospective cohort of ICU-treated COVID-19–related ARDS, cumulative 4-year all-cause mortality remained substantial, reaching 45%. In exploratory multivariable analyses, older age and higher white blood cell count at ICU admission were consistently associated with both early (30-day) and late mortality, suggesting that basic patient factors and inflammatory burden capture an important part of the prognostic signal observed in more complex models. Among 4-year survivors who completed telephone follow-up, a considerable proportion reported persistent symptoms and limitations, including sleep disturbance, fatigue, dyspnoea, cognitive complaints, and reduced functioning, with measurable consequences for health-related quality of life and return to work. Reported rehabilitation uptake was relatively low, and the association between rehabilitation and lower QALY likely reflects confounding by indication rather than a harmful effect of therapy. Finally, the cumulative burden of post-ICU impairments showed a graded relationship with lower long-term QALY, underscoring the need for structured, multidisciplinary follow-up pathways and improved access to tailored rehabilitation and socioeconomic support for ICU survivors.

The datasets generated and/or analyzed during the current study are not publicly available due to legal and ethical restrictions related to patient confidentiality and GDPR compliance. De-identified data may be made available from the corresponding author upon reasonable request, subject to appropriate ethical approvals and data-sharing agreements.

No specific grant from any funding agency, commercial, or not-for-profit sectors was received for this study. Educational support for manuscript preparation was provided by the Medical Research Agency (ABM, Warsaw, Poland) through participation in the Harvard Medical School Polish Clinical Scholars Research Training Program.

## Supplementary Information

Below is the link to the electronic supplementary material.

Supplementary Material 1

## Data Availability

The datasets generated and/or analyzed during the current study are not publicly available due to legal and ethical restrictions related to patient confidentiality and GDPR compliance. De-identified data may be made available from the corresponding author upon reasonable request, subject to appropriate ethical approvals and data-sharing agreements.
