# Supplementary material for: Four year mortality and quality of life after ICU treatment for COVID 19 related acute respiratory distress syndrome
Source: Sci Rep. 2026 Mar 1;16:11510. doi: 10.1038/s41598-026-42341-1 (PMC13057305; doi:10.1038/s41598-026-42341-1)
Supplement: Supplementary file 1 — Supplementary Material 1 [file 41598_2026_42341_MOESM1_ESM.pdf]

Supplementary materials

Figure S1. Forest plot of adjusted odds ratios (OR) for 30-day mortality predictors

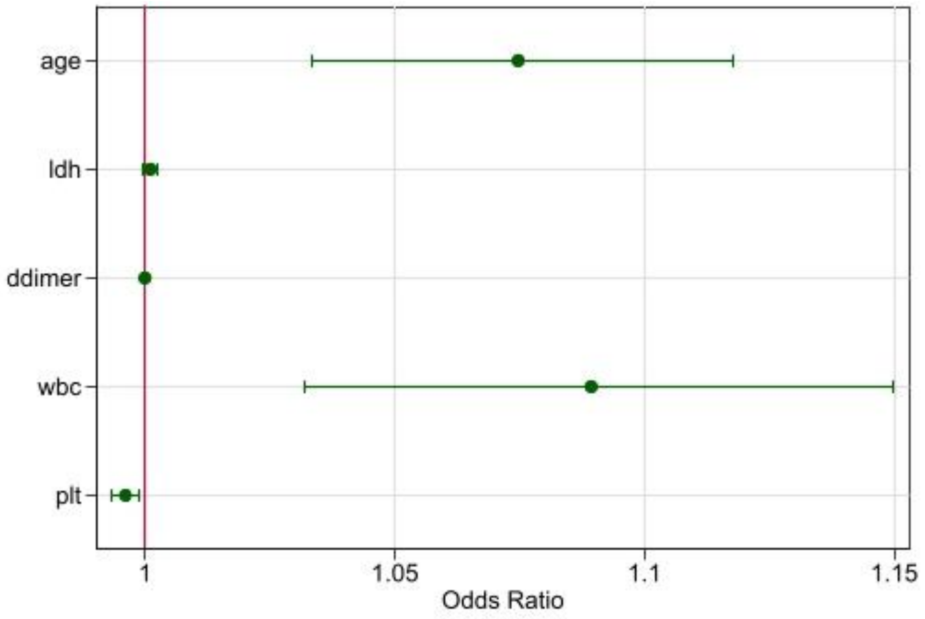

Forest plot of adjusted odds ratios (ORs) for 30-day mortality based on a multivariate logistic regression model including age, LDH, D-dimer, WBC, and PLT. Error bars represent 95% confidence intervals.

Figure S2. Forest plot of adjusted odds ratios (OR) for the long-term mortality predictors.

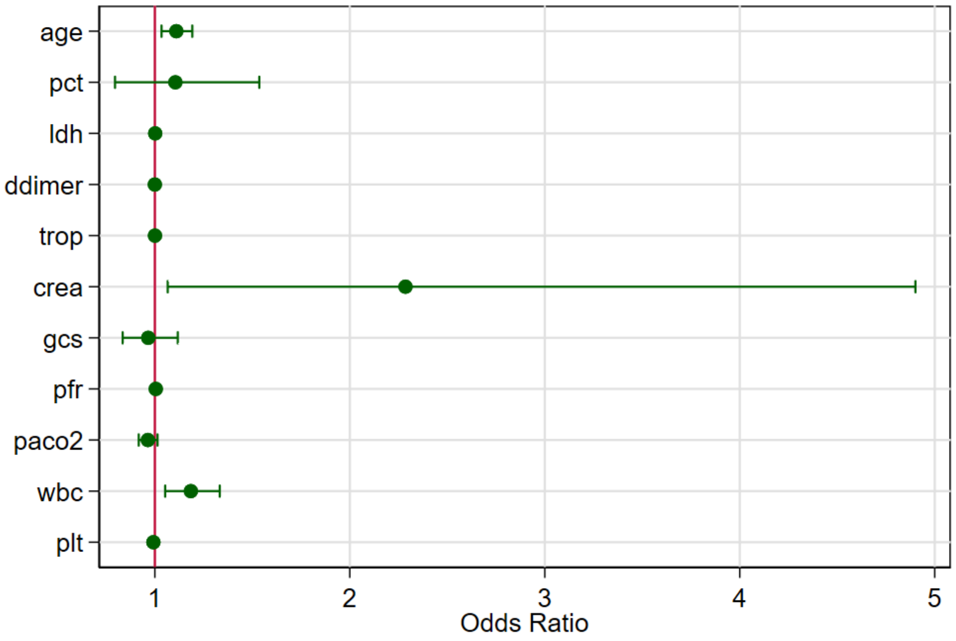

Forest plot of adjusted odds ratios (OR) for long-term (30-day - 4-year) mortality among ICU survivors. The multivariate logistic regression model included Age, lactate dehydrogenase (LDH), D-dimer, Troponin, Creatinine, Glasgow Coma Scale (GCS), PaO2/fiO2 (PFR), partial pressure of carbon dioxide in arterial blood ($PaCO_2$), white blood cell count (WBC), procalcitonin (PCT), platelet count (PLT). Error bars represent 95% confidence intervals. Age, WBC, and Creatinine were independently associated with increased risk of long-term mortality.

Figure S3. Forest plot of adjusted odds ratios (OR) for factors associated with lower QALY at 4 years.

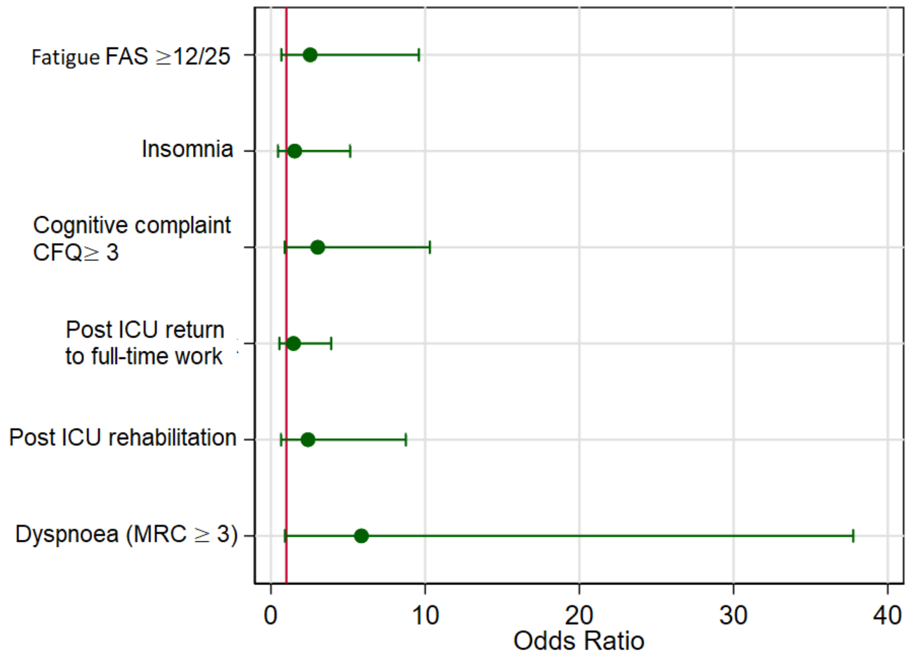

Forest plot showing results from a multivariable logistic regression model examining factors associated with lower quality-adjusted life years (QALY) at 4-year follow-up.

Figure S4. ROC curve for the 30-days mortality prediction model

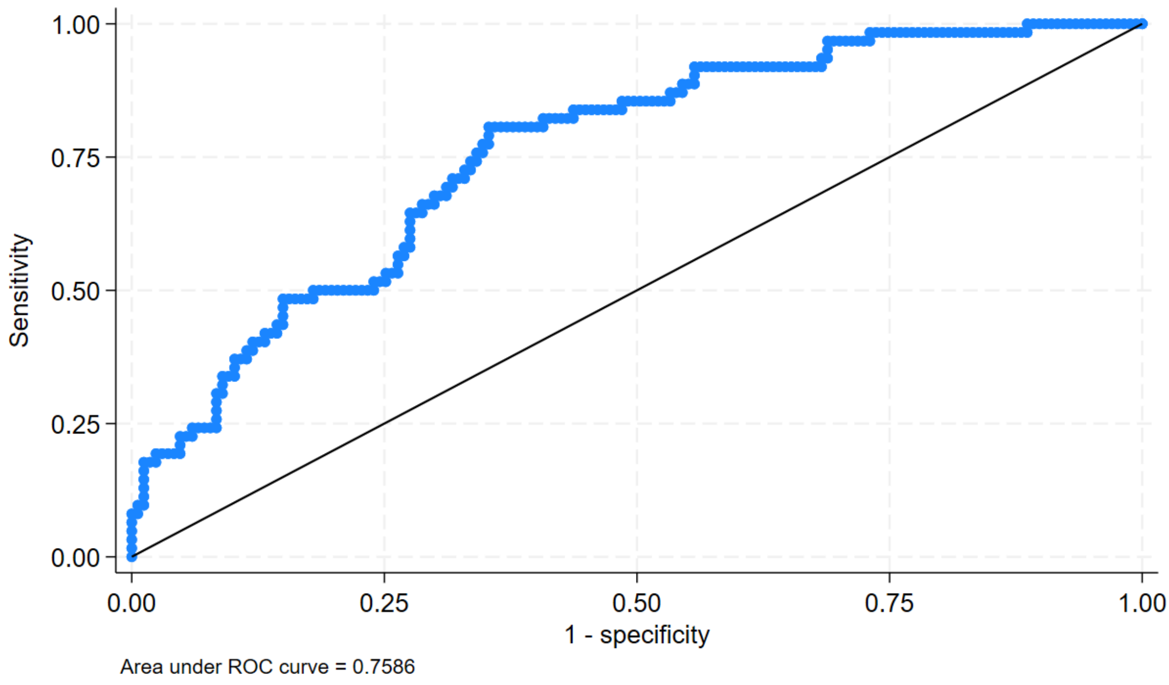

Receiver operating characteristic (ROC) curve for the exploratory multivariable logistic regression model predicting 30-day mortality. The model demonstrated higher apparent discrimination (AUC 0.76, 95% CI 0.69–0.83) but included multiple correlated laboratory variables and should be interpreted as exploratory.

Table S1. Baseline characteristics – Responders v.s. Lost to telephone follow-up.

Variable<br><br>Continous	Responders to follow-up<br>n = 81<br><br>Mean ± SD<br><br>Median [IQR]*	Lost to follow-up<br>n = 77<br><br>Mean ± SD<br><br>Median [IQR]*	p-value
WBC (10^3/µL)	9.7 ± 4.4	11 ± 5.5	0.11
LDH (u/L)	573.5 ± 180.2	600.1 ± 265.9	0.48
Age (years)	56.7 ±11.6	55.8 ± 9.3	0.58
BUN (mg/dL)*	18.2 [14–26]	20.3 [16.2– 26]	0.08
Ferritin (ng/mL)*	1564.9 [774-2000]	1192.9 [617.2-2000]	0.15
Creatinine (mg/dL)	0.89 ± 0.43	1.03 ± 0.82	0.18
**Binary**	**n (%)**	**n (%)**	
Coronary heart disease	2 (2.5)	6 (7.8)	0.13
Chronic kidney disease	0 (0)	1(1.3)	0.31
Female sex	24 (30)	31 (40.3)	0.18
Current smoker	4 (5)	6 (7.8)	0.47
Arterial hypertension	38 (47.5)	43 (55.8)	0.29
Type 2 diabetes	9 (11.3)	17 (22.1)	0.07

Baseline demographic and clinical characteristics of 4-year survivors according to follow-up status. Responders were defined as patients who completed the structured telephone interview, while non-responders were alive at 4 years but did not participate. Variables are presented as mean (standard deviation), median [interquartile range], or number (%), and compared using the Student's *t*-test, Wilcoxon rank-sum test, or chi-squared test, as appropriate.
